# Bichloride of Mercury and Chloride of Zinc in the Treatment of Pulpless Teeth and Alveolar Abscess

**Published:** 1893-08

**Authors:** Frank Abbott

**Affiliations:** New York


### International Dental Journal.

Vol. XIV. August, 1893. No. 8.

### Original Communications.[1]

### BICHLORIDE OF MERCURY AND CHLORIDE OF ZINC IN THE TREATMENT OF PULPLESS TEETH AND ALVEOLAR ABSCESS.[2]

BY PROFESSOR FRANK ABBOTT, NEW YORK.

Mr. President and Gentlemen of the New York Odontological Society,—The subject to which I will ask your attention for a few minutes this evening is, in one sense, old and hackneyed, but one full of interest to every practitioner of dental surgery.

There have been so many methods of treatment, and so many remedies recommended, that it almost seems that time spent in a further consideration of the subject would be thrown away. I hope, however, to not be voted guilty of occupying your time this evening for nothing.

If there are pathological conditions to be found in the human organism of more interest, and those which demand a more careful study and more thorough and scientific treatment, than those connected with the oral cavity, I have yet to become acquainted with them. In the treatment of pulpless teeth the results are, I believe, unique in the practice of medicine. That living and dead tissue can be made to exist in the same organ in the system without

---

[1] The editor and publishers are not responsible for the views of authors of papers published in this department, nor for any claim to novelty, or otherwise, that may be made by them. No papers will be received for this department that have appeared in any other journal published in the country.

[2] Read before the New York Odontological Society, May 16, 1893.

presenting to either the patient or the practitioner any special pathological condition, is an achievement in medical and surgical practice of which dentists have a just right to be proud.

It is now some thirty years since it was first believed that a tooth which had been neglected or unfortunate enough in any way to have lost the life of its nourishing organ could be so treated as to become not only useful, but rendered in a condition so nearly physiological as to give the patient no annoyance for many years, perhaps for life.

It will be remembered by the older gentlemen present that the late Dr. Wm. H. Atkinson was the first (at about the time mentioned) to assert that pulpless teeth, with or without abscesses, could be successfully treated and made useful and comfortable organs to the patient forever after. You probably also remember the elaborate and long-continued treatment that he followed and taught. Again, you perhaps remember that the writer took issue with him upon his methods of treatment of these cases as well as upon several other modes of practice. From that time to the present there has been very little difference of special importance published in the methods of treatment except as regards the remedies used, and the manner of applying them. Every practitioner, as far as I know, with few exceptions, possibly still adheres to the practice of more or less continued treatment of pulpless teeth before venturing to fill them. That this is a mistake, fraught with a great amount of uncertainty and trouble to the dentist and long-continued annoyance and expense to the patient, it is my purpose to show you this evening.

A saturated solution of iodine in creosote was, I believe, the first remedy claimed to possess the necessary properties for the successful treatment of either of the varieties of teeth under consideration. This remedy, as many of you probably remember, was a very disagreeable mixture to handle. The patient's breath, the operator's hands, instruments, and in fact everything with which it came in contact, became loaded with its unpleasant odor, which nothing seemed capable of eradicating, and the results obtained with it were, in most cases, anything but satisfactory. Then followed in quick succession carbolic acid and creosote. These were, if anything, less satisfactory in their results than the first. The odor of carbolic acid is not so disagreeable nor so lasting as the first mentioned remedy, but it, like that, failed in what was expected of it. When applied undiluted to animal tissue they are both quite powerful potential cauteries, and undoubtedly antiseptic.

The odor of creosote is less objectionable. Pure beechwood creosote is rather pleasant than otherwise to many persons to both the organs of taste and smell, but as a remedy in the treatment of pulpless teeth it is not all that could be desired. When applied undiluted, like the others, it is a good mild potential cautery, and its antiseptic properties are said to be far superior to that of carbolic acid. Are not all of these remedies, however, soon absorbed, and the original conditions, so to speak, re-established? Admitting, if you please, that by the use of these substances all organisms are destroyed, judging from the results it would seem that something was left intact, which causes either the continuance of the trouble or the beginning of new and troublesome complications. There is, in my judgment, something more to be accomplished in the treatment of pulpless teeth than the death of organisms of decomposition. The result of the putrefactive process—the ptomaines—must be rendered inert, otherwise it is fair to presume that the putrefactive process will continue.

It is, I believe, a well-established fact in the minds of most pathologists that any substance, claimed to be a germicide, is much more effective when greatly diluted than in its pure state. This is due, probably, to two facts: first, the diluted remedy is more readily absorbed by the diseased or dead tissue, the parts becoming almost immediately, upon its application, saturated with it, while the pure substance is absorbed very slowly; secondly, in the process of imbibing nourishment the organisms take the diluted remedy because there is no uncontaminated nourishment at hand for them to live upon. Consequently they take this and die rather than die of starvation. Thus it is that a solution of carbolic acid of 1 to 100 is classed as an effective germicide; creosote, 1 to 200, etc.

Many remedies have been introduced to the medical profession of more or less value as antiseptics, and with which good results are accomplished, but none of them are considered to-day as equal in destroying germ-life and arresting putrefaction to bichloride of mercury.

One special advantage this remedy possesses is its ability to accomplish the results desired in so greatly a diluted state. It is claimed to be a germicide when diluted to 1 to 100,000 parts of water. To use it, however, so weak in the pulp-canals of teeth would probably prove unsatisfactory, in consequence of the presence of more or less liquid in the canals, which would further dilute it and render its action *nil*. Another great advantage in its use lies in its odorless quality. A solution of 1 to 10,000 of water is almost as tasteless and odorless as the water alone, and still this strength will do all that is desired, and may be used freely in the teeth, with no possible harm to the patient. At the same time it is strong enough, even if it is more or less diluted by the liquid contents of the canals, to do the work desired of it. From the almost positive results in the use of this remedy in the treatment of pulpless teeth, I am led to believe that it not only destroys all organisms, but renders ptomaines, if any are left in pulp-canals, harmless. This, I take it, is the result of a chemical change that takes place when the solution of bichloride and the ptomaines meet, rendering the ptomaines inert. The method practised in the treatment of these cases, where no visible abscess is present opening upon the gum, and to which I wish especially to call your attention, is as follows:

After the contents of the pulp-canals are stirred and all removed that can be with an instrument that plays freely in them, a solution of 1 to 10,000 of bichloride of mercury is thrown into them by means of a small gold-pointed (Farrar) syringe. This stirring and washing is repeated until the operator is satisfied as to the cleanliness of the canals; then, in most cases,—not in all,—a very small bit of cotton is carried to the distal end of the canal and packed tightly, plugging the foramen.

It will be observed that up to this stage of the operation, when everything is ready for the introduction of the filling-material, the canals have not been *drilled out*, nor any attempt made to dry them; the cavity in the crown and the pulp-chamber are dried, but the canals proper have been made aseptic, and the antiseptic is allowed to remain and mix with the filling-material, which is immediately introduced into the canals (unless periostitis is present, when, if it is, a day or two of treatment will usually relieve it).

The filling-material consists of oxychloride of zinc, with one drop of a solution of 1 to 2000 of bichloride of mercury mixed with it. The final operation upon the crown of the tooth is immediately done and finished. The addition of the bichloride solution to the cement is made in order that the effect of its constant presence may be assured. As a precaution, the gum over the tooth is then painted with "antiphlogistine" (concentrated tincture of aconite root and tincture of iodine, equal parts). This mode of treatment has been followed by the writer altogether for some two or three years, and in some cases it was done by him as long ago as seven or eight years. It was the success in the treatment of these cases, and a careful study of the subject, that has resulted in adopting the method altogether.

In many cases, I might say nearly all, when the canals are being filled, the filling-material or the liquid (chloride of zinc) is forced through the foramen, and, coming in contact with the living tissue at the end of the root, produces, through its escharotic action, slight pain, which usually lasts from fifteen minutes to an hour.

Let us consider for a moment what advantages are gained by this over that of the protracted treatment so generally followed.

If a pulp-canal is left open and *treated* occasionally, the gas that generates in the process of putrefaction of the contents of the dental canaliculi must escape into it, as it has no other exit. The canal very soon becomes filled with it, so that both mechanically and chemically it acts upon and irritates the living tissue at the end of the root of the tooth, so that every day sees the case complicated with perhaps pain, perhaps suppuration and discharge of pus through the canal. This condition may, and in many cases does, continue for days, weeks, and sometimes even for months before it seems at all safe to fill it. All this is obviated in the treatment above described, consequently it should commend itself to all dental practitioners.

Should any perisosteal disturbance follow the operation, by again painting the gum once or twice with the above-mentioned remedy it subsides, and the tooth becomes as comfortable as it was before exposure of the pulp. Such trouble, however, seldom occurs, the one painting before the patient leaves the chair usually being all that is required.

The treatment of teeth with abscesses is proceeded with in the same general way as far as cleansing and the use of the bichloride is concerned. After this has been thoroughly done, a solution of twenty grains of chloride of zinc to one ounce of water in ordinary cases (forty to sixty grains in chronic cases) is forced through the canal and abscess until its presence upon the gum is indicated by the characteristic white coagulated albumen. The canals and crown are then filled and finished as before described, except that no cotton is placed in the canal before introducing the oxychloride mixture.

The advantages gained by this mode of treating alveolar abscesses over that so generally practised must be apparent to every practitioner.

The chloride of zinc possesses properties which no other remedy does that I am familiar with, that make it one of the most valuable, if not *the* most valuable, agent known in the treatment of this troublesome disease. It mingles or mixes freely with the liquid contents of the abscess, does its work (destroys the lining membrane of the sac) with very little pain, and its powerful astringent properties, together with its highly-stimulating effect, serve to almost immediately produce healthy granulations, which in a few days results in closing the cavity at the end of the root and the healing of the opening in the gum.

I am aware of the confidence many practitioners reposed in carbolic acid, creosote, iodoform, and other escharotic and antiseptic agents in the treatment of alveolar abscesses. I used them nearly all for many years, nearly always with unsatisfactory results. The lack of the necessary escharotic properties, due, perhaps, in a great measure to the fact that they became too much diluted upon entering the abscess proper, by mixing with its liquid contents, thus rendering them of little, if any, value as a destroyer of the lining membrane (pyogenic membrane) of the abscess. Consequently, the sac remains to receive the migrating, colorless blood-corpuscles which constitute, after their death and partial decomposition, the pus which is constantly or periodically discharging through the opening in the gum.

The chloride of zinc, on the other hand, even if somewhat diluted, takes hold of this abscess-sac so vigorously that its lining membrane is almost immediately destroyed, and it will in a short time (two or three days) break down, and discharge in the form of pus.

Should the canals be left open after its use, or that of any other remedy, the gas from the putrefactive process going on in the canaliculi will very shortly re-establish the abscess, so that the treatment must be repeated over and over again, each time with varying degrees of success or failure.

Alveolar abscesses differ from those in soft tissue in that there is no repair taking place, even after evacuation. The bony walls being unyielding, and not containing sufficient organic tissue to exert pressure upon the abscess, and thus cause its obliteration, it remains, in most cases, for many years, or until the tooth is removed or an escharotic strong enough to destroy it is forced into it. Antiseptics, consequently, are of little use, except to prepare the parts for the more perfect action of the escharotic.

